# Effects of semaglutide on cardiovascular risk factors and eating behaviors in type 2 diabetes

**DOI:** 10.1007/s00592-022-01936-6

**Published:** 2022-07-17

**Authors:** Ugo Di Folco, Noemi Vallecorsa, Maria Rosaria Nardone, Angelo Lauria Pantano, Claudio Tubili

**Affiliations:** Diabetes Unit, “S. Camillo-Forlanini” Hospital, Rome, Italy

**Keywords:** Type 2 diabetes, Semaglutide, Effectiveness, Treatment satisfaction, Eating behaviors HbA1c, Weight, Hyperglycemia, Non-HDL cholesterol

## Abstract

**Aims:**

Aim of the present study was to evaluate the impact of once-weekly semaglutide on different end-points indicative of metabolic control, cardiovascular risk, dietary behavior, and treatment satisfaction in T2DM.

**Methods:**

This was a retrospective observational study conducted in a diabetes clinic. Changes in HbA1c, fasting blood glucose (FBG), weight, blood pressure, lipid profile, and number of antihypertensive drugs at 32 weeks (T1) after the first prescription of semaglutide (T0) were analyzed. Furthermore, at T1 patients were asked to fill-in the Diabetes Treatment Satisfaction Questionnaire (DTSQ) and the Control of Eating Questionnaire (COEQ).

**Results:**

Overall, 104 patients were identified (mean age 63.6 ± 10.4 years, 58.7% men, diabetes duration 12.7 ± 8.7 years). After 32 weeks of treatment with semaglutide, HbA1c levels were reduced by 1.38%, FBG by − 56.53 mg/dl, weight by 6.03 kg. Systolic and diastolic blood pressure, total, HDL-, LDL-, and non –HDL cholesterol, and triglycerides significantly improved. The number of glucose-lowering and antihypertensive drugs also decreased. At T1, DTSQ score was 32.23 ± 1.44, whereas COEQ indicated low levels of hunger and good control of eating.

**Conclusions:**

The study documented benefits of semaglutide on metabolic control and multiple CV risk factors, simplification of therapeutic schemes and high satisfaction with diabetes treatment, and eating behaviors indicative of healthy diet and reduced food intake.

## Introduction

Type 2 diabetes mellitus (T2DM) is a metabolic disease characterized by hyperglycemia caused by insulin resistance and deficiency of insulin secretion by the beta cells of the pancreas [1]. In the last decade, glucagon-like peptide receptor agonists (GLP1-RAs) have been approved as a new therapeutic option in T2DM. These drugs mimic the action of endogenous GLP-1, a hormone produced by enteroendocrine L cells following the ingestion of nutrients, especially carbohydrates [2]. Its main function is to increase the secretion of insulin by pancreatic *β* cells in a glucose-dependent manner and to inhibit the secretion of glucagon by *α* cells. GLP1-RAs also preserve the function of *β*-cells, stimulating their proliferation and differentiation and inhibiting apoptosis [3]. GLP-1 receptors are also present in the gastrointestinal tract, in the cardiovascular system, and in the central nervous system, in particular in the nucleus of the solitary tract; these mediate a slowdown in gastric emptying, with an increase in the sense of satiety reduced hunger and lower energy intake [4, 5]. In animal models, data suggest that these effects may be due to GLP-1 acting directly on receptors in the brain, affecting perceptions of the reward value of food [6]. Due to their mechanisms of action, GLP1 RAs reduce body weight and blood pressure levels [7].

In most patients with T2DM the lipid profile is altered with hypertriglyceridemia, increased total cholesterol and low-density lipoproteins (LDL) cholesterol levels, and decreased high-density lipoproteins (HDL) cholesterol levels. All of these alterations constitute the “atherogenic dyslipidemia in diabetes” which contributes to the increase in cardiovascular risk typical of subjects with T2DM [8]. The non-high-density lipoproteins (non-HDL) cholesterol allows a better estimate of the cardiovascular risk, because it represents the cholesterol of all atherogenic particles, such as LDL, lipoprotein A, very high-density lipoproteins low (VLDL) and intermediate density lipoproteins (IDL) [9, 10]. Non HDL cholesterol represent a prevalent CV risk factor in T2DM [11, 12], but no data are available about the impact of GLP1-RA on it.

Semaglutide is an analogue of native GLP-1 with a prolonged half-life (165 h), suitable for administration once a week (ow). Semaglutide was shown to be effective in improving metabolic control in T2DM by reducing blood glucose and glycated hemoglobin (HbA1c), body weight, and cardiovascular risk [13].

The side effects that may occur are nausea and, more rarely, vomiting and diarrhea, usually mild and limited to the first weeks of treatment [14].

Aim of the present study was to evaluate the impact of semaglutide on different end-points indicative of metabolic control, cardiovascular risk, dietary behavior, treatment satisfaction in T2DM, and a possible reduction in the use of antihypertensive drugs.

## Methods

This was a retrospective observational study conducted in the diabetes clinic of S. Camillo Forlanini Hospital–Rome. Data on all patients treated with ow semaglutide were extracted from the electronic medical record system adopted in the hospital.

Baseline visit was represented by the first prescription of semaglutide (T0); follow-up visits were based on routine clinical practice, usually every 4 months. Values recorded after 32 weeks from baseline were used for this study (T1).

According to the summary of product characteristics, all patients were first prescribed with semaglutide 0.25 mg; semaglutide was then titrated up to 0.50 mg and successively 1.0 mg (based on patient needs).

Baseline characteristics were collected including: age, gender, diabetes duration, obesity indices (BMI, weight), smoke, HbA1c, fasting blood glucose (FBG), systolic blood pressure (SBP), diastolic blood pressure (DBP), lipid profile (total, LDL, HDL, non HDL cholesterol, and triglycerides), estimated glomerular filtration rate (eGFR), albuminuria, micro- and macro-vascular diabetes complications, comorbidities, diabetes treatment before and at initiation of semaglutide, and antihypertensive treatment.

At follow-up visit, data on changes in HbA1c, FBG, BMI, body weight, blood pressure, lipid profile, number of antihypertensive drugs were collected.

Furthermore, at T1 patients were asked to fill-in the Diabetes Treatment Satisfaction Questionnaire (DTSQ) [15, 16] and a questionnaire on eating habits (Control Of Eating Questionnaire, COEQ) [17, 18. Italian versions of these questionnaires were administered.

DTSQ has been specifically designed to measure satisfaction with diabetes treatment regimens [15]. It is composed of eight items, six of which are summed in a single score ranging from 0 (very dissatisfied) to 36 (very satisfied). The remaining two items are treated individually and explore the perceived frequency of hyperglycemic and hypoglycemic episodes, with higher scores indicating a higher frequency. The Italian version of the instrument has been previously translated and validated [16].

Control of eating and the degree of food cravings were measured using a modified version of the validated 16-item short form COEQ, which includes questions related to food cravings, control of eating, hunger and fullness. Based on the previous 7 days, subjects were asked to rate the first 14 questions of the original COEQ on a 10 cm visual analogue scale (VAS); 1 question with categorical response was added to investigate about type of food least liked by the patient [17, 18].

The study protocol was approved by the local ethics committee. Informed consent was obtained from all patients for being included in the study.

In Italy, GLP1-RA therapy is reimbursed by the national healthcare system in all patients with T2DM and HbA1c > 7.0% as a monotherapy (when metformin is contraindicated or not tolerated) or in combination with other antihyperglycemic agents.

### Statistical analysis

Considering the preliminary, descriptive nature of this study, a formal sample size calculation was not performed. However, a minimum sample size of 47 patients allowed to detect with a statistical power of 90% a decrease in HbA1c levels of at least 0.5%, assuming an estimated standard deviation of differences of 1.0 and with a significance level (alpha) of 0.05.

Descriptive data were summarized as mean and standard deviation for continuous variables or frequency and proportion for categorical variables.

Changes in continuous study endpoints were assessed using mixed models for repeated measurements. Results are expressed as estimated mean or estimated mean difference from T0 with their 95% confidence interval (95% CI). Paired t-test derived from linear mixed models for repeated measurements were applied for within group comparisons. Statistical significance was declared if *p*-value was < 0.05.

## Results

Overall, 104 patients receiving the first prescription of semaglutide between January and March 2021 were identified through electronic medical records adopted in the clinic. Baseline patient characteristics are reported in Table 1. Mean age was 64 years, 58.7% were men, diabetes duration was of 12.7 ± 8.7 years.

**Table 1:** Baseline patient characteristics

	Mean and standard deviation or proportion
N	104
Age (years)	63.6 ± 10.4
Men (%)	58.7
Diabetes duration (years)	12.7 ± 8.7
BMI (Kg/m^2^)	32.9 ± 5.9
Smokers (%):	
No	66.3
Yes	26.9
Ex	6.7
HbA1c (%)	8.5 ± 1.8
Fasting blood glucose (mg/dl)	186.0 ± 60.0
Systolic blood pressure (mmHg)	135.8 ± 12.8
Diastolic blood pressure (mmHg)	79.6 ± 8.3
Total cholesterol (mg/dl)	190.3 ± 46.1
LDL cholesterol (mg/dl)	105.5 ± 39.5
HDL cholesterol (mg/dl)	44.8 ± 12.3
Non HDL cholesterol (mg/dl)	145.5 ± 44.7
Triglycerides (mg/dl)	200.0 ± 118.4
Hypertension (%)	61.2
Dyslipidemia (%)	100.0
Retinopathy (%)	9.6
Organ damage (%)*	51.0
Myocardial infarction (%)	10.6
Stroke (%)	2.9
Coronary rivascularization (%)	9.6
Heart failure (%)	2.9
eGFR < 60 ml/min*1.73 m2	5.8
Albuminuria > 300 mg/dl	1.0

*Myocardial infarction, stroke, limb/feet amputation, coronary or peripheral revascularization, heart failure, carothid or peripheral vessels plaques, retinopathy, kidney damage markers (eGFR < *60, albuminuria* > *300 mg/die, haemodialysis, kidney transplantation*

Metabolic control was poor (Fasting Blood Glucose186.0 ± 60.0 mg/dl; HbA1c 8.5 ± 1.8%).

At T0, patients showed poor metabolic control, high mean BMI, and poor control of cardiovascular risk factors. Furthermore, organ damage was recorded in one half of patients. Before starting semaglutide, 6 (5.8%) patients were already treated with another GLP1-RA. Furthermore, 98 (94.2%) patients were treated with oral hypoglycemic agents (OHA), of whom 72 (69.2%) with 1 OHA and 26 (25.0%) with  ≥ OHAs. Moreover, 11 (10.6%) patients were treated with schemes including insulin.

At initiation of semaglutide, 20 (19.2%) patients were treated with semaglutide only, 79 (76.0%) patients were treated with OHAs, of whom 75 (72.1%) with 1 OHA and 4 (3.9%) with 2 OHAs, and 8 (7.7%) patients were treated with schemes including insulin.

After 32 weeks of treatment with semaglutide, HbA1c levels were reduced by 1.38% and all continuous endpoints showed statistically significant and clinically relevant improvements (Table 2).

**Table 2:** Changes in estimated mean levels of continuous clinical endpoints over time

Change in	Visit	Estimated mean and 95% CI	Estimated mean difference from T0 and 95% CI	*p*-value
HbA1c (%)	T0	8.55(8.27;8.83)		
	T1	7.16(6.88;7.44)	− 1.38 ( − 1.68; − 1.09)	** < 0.0001**
FBG (mg/dl)	T0	186.03 (177.05;195.01)		
	T1	129.5 (120.52;138.48)	− 56.53 ( − 67.17; − 45.89)	** < 0.0001**
BMI (Kg/m^2^)	T0	32.9 (31.81;33.99)		
	T1	30.73 (29.63;31.82)	− 2.18 ( − 2.56; − 1.79)	** < 0.0001**
Weight (Kg)	T0	94.23 (90.75;97.72)		
	T1	88.2 (84.72;91.69)	− 6.03 ( − 7.13; − 4.93)	**< 0.0001**
SBP (mmHg)	T0	135.82 (133.63;138.01)		
	T1	133.06 (130.87;135.25)	− 2.76 ( − 4.08; − 1.44)	** < 0.0001**
DBP (mmHg)	T0	79.57 (78.05;81.08)		
	T1	75.72 (74.21;77.23)	− 3.85 ( − 5.02; − 2.67)	** < 0.0001**
Total chol (mg/dl)	T0	190.33 (181.44;199.22)		
	T1	172.18 (163.29;181.07)	− 18.14 ( − 23.99; − 12.3)	** < 0.0001**
HDL-chol (mg/dl)	T0	44.84 (42.49;47.18)		
	T1	47.44 (45.1;49.79)	2.61 (1.14;4.07)	**0.0006**
LDL-chol (mg/dl)	T0	105.49 (97.87;113.12)		
	T1	94.56 (86.94;102.19)	− 10.93 ( − 16.47; − 5.39)	**0.0002**
TG (mg/dl)	T0	199.99 (181.44;218.54)		
	T1	150.89 (132.35;169.44)	− 49.1 ( − 65.47; − 32.72)	** < 0.0001**
Non HDL-chol (mg/dl)	T0	145.49 (137.11;153.87)		
	T1	124.74 (116.36;133.12)	− 20.75 ( − 26.71; − 14.79)	**< 0.0001**

*HbA1c* Glycated haemoglobin, *FBG *Fasting blood glucose, *SBP* Systolic blood pressure, *DBP* Diastolic blood pressure, *chol *Cholesterol, *HDL* High-density lipoprotein, *LDL* Low-density lipoprotein, *TG* Triglycerides, 95% CI = 95% confidence intervals

*Values in bold are statistically significant

At the end of the observation, 100% of patients were treated with 0.5 mg.

**Fig. 1:**
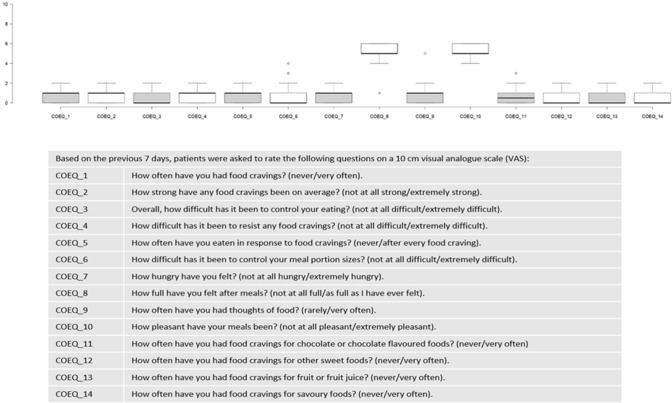
Control of eating questionnaire (COEQ)

**Fig. 2:**
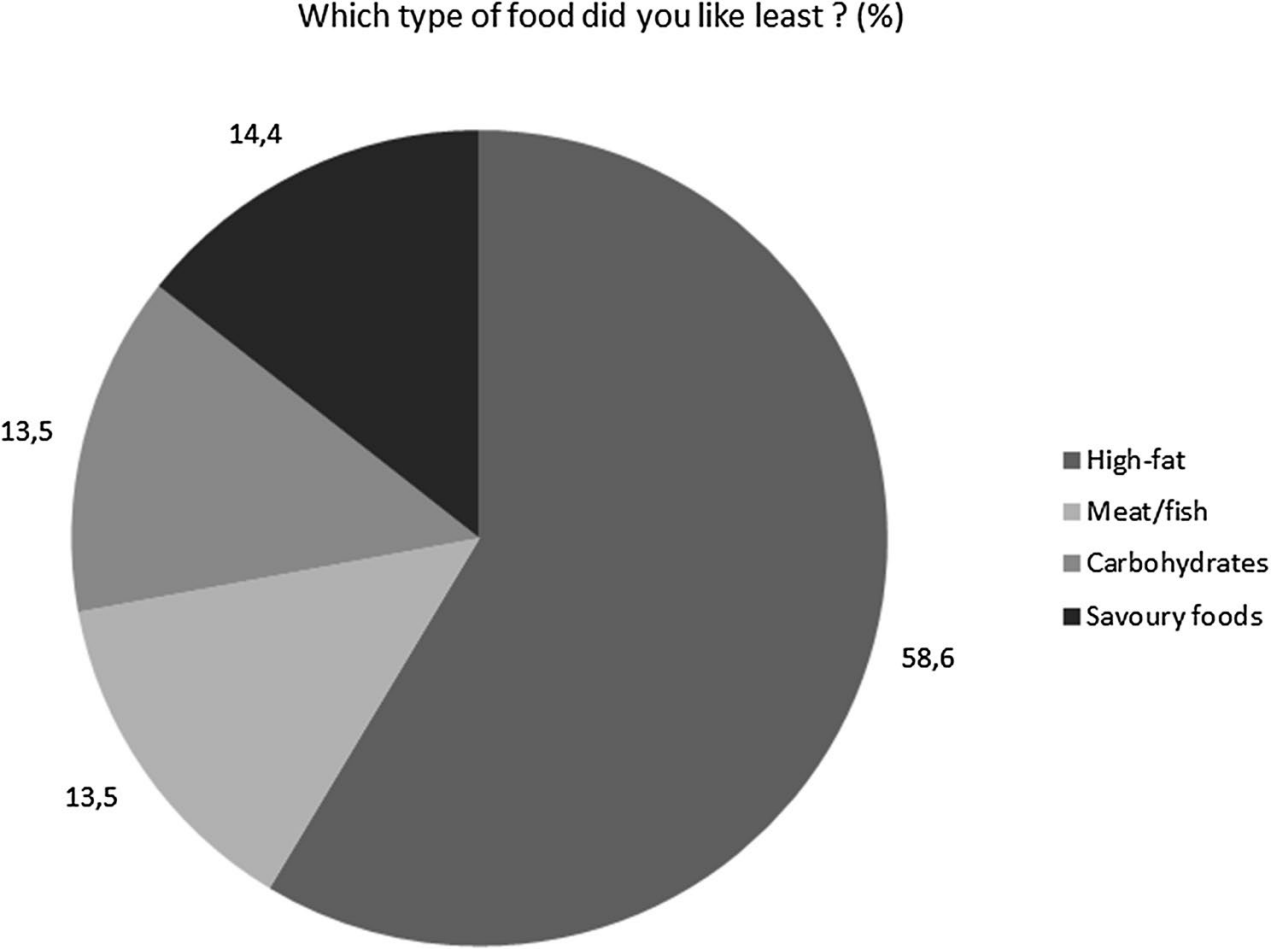
Control of eating questionnaire (COEQ)

The COEQ items at T1 indicated low levels of hunger, good control of eating and meal portion size, and low levels of food cravings, with most of items reaching a median VAS value between 0 and 1. Intermediate levels of pleasantness and fullness after meals were registered, with items reaching a median VAS value of around 5 (Fig. 1). The last item indicated the lowest liking for high-fat foods in 58.7% of patients (Fig. 2).

DTSQ score at T1 was close to the maximum value (median value 33, interquartile range 32-34).

As for the use of antihypertensive drugs, at T0, 40 patients (38.8%) were not treated with antihypertensive drugs, while 34 (33.0%), 24 (23.3%), and 5 (4.9%) were treated with 1, 2, 3 antihypertensive drugs, respectively. At T1, 39 patients (37.5%) were not treated with antihypertensive drugs, while 38 (36.5%), 23 (22.1%), and 4 (3.8%) were treated with 1, 2, 3 antihypertensive drugs, respectively.

Overall, 17 out of 104 (16.3%) patients reported side effects, of whom 13 (12.5%) nausea and 4 (3.8%) abdominal bloating. These symptoms were generally mild and decreased over time. Two patients discontinued the treatment with semaglutide during 32-week follow-up. No episode of severe (requiring third part assistance) or clinically relevant (blood glucose < 54 mg/dl) hypoglycemia occurred.

## Discussion

### Main findings

This study documented the effectiveness and tolerability of semaglutide in patients with uncontrolled T2DM.

After 32 weeks of treatment, HbA1c levels and body weight significantly decreased ( − 1.38% and − 6.03 kg, respectively) and the entity of the reduction was in line with results obtained in the SUSTAIN program [13, 19, 20], where HbA1c was reduced from 1.1 to 1.5% with semaglutide 0.5 mg and from 1.4 to 1.8% with semaglutide 1 mg; weight was reduced from 3.5 to 4.6 kg with semaglutide 0.5 mg and from 4.5 to 6.5 kg with semaglutide 1 mg. In addition, real-world studies conducted in US and Europe confirmed the effectiveness and safety of semaglutide when administered under routine clinical practice conditions, although the magnitude of benefits varied based on patient profiles and settings [21–27].

In addition to the effect on HbA1c and weight, in our study we documented improvements in lipid profile and blood pressure.

Finally, our population presented multiple associated risk factors (dyslipidemia, hypertension, obesity), 50% had organ damage and 23.1% had history of cardiovascular event.

### Comparison with existing knowledge

The recent documentation of the positive CV effects of semaglutide in reducing the MACE risk [13] is extremely encouraging in relation to the clinical use of this drug. Furthermore, a post-hoc analysis of the LEADER and SUSTAIN 6 CV outcome trials confirmed the importance of addressing multiple risk markers in T2DM to reduce CV and renal risk, therefore stressing the importance of multifactorial interventions targeting all risk markers [28].

Following these findings, in July 2021 new AMD-SID (Associazione Medici Diabetologi; Societa’ Italiana di Diabetologia) Italian Guidelines stated that GLP1-RA can be prescribed as a first line therapy in T2DM with a previous cardiovascular event. ADA Guidelines (ADA 2022) also suggested to consider GLP1-RA as a first choice therapy in high cardiovascular risk patients [29, 30].

In our study population and in general T2DM population, obesity is present in the majority of patients and its role as main driver of the disease is well known [31]. Weight loss is one of the most important therapeutic goals and is associated with an improvement of all the cardiovascular risk factors – cholesterol, tryglicerides, blood pressure—and with an improvement of cardiac function and overall prognosis (ESC 2021) [32]. GLP1-RAs were the first glucose-lowering drugs that induce weight loss [33–35] due to their pleiotropic actions including a central nervous system interaction with reward circuits and food intake. Semaglutide effect was demonstrated to be superior to that of dulaglutide (SUSTAIN 7) and liraglutide (SUSTAIN 10) in T2DM at therapeutic doses [36, 37].

Semaglutide produces significant benefits on cardiovascular risk, as demonstrated in SUSTAIN 6, where a significant lower rate vs. placebo of 3 points MACE was documented [13]. The reduction of CV risk is mediated by improvements in risk factors. In our study, total cholesterol level significantly decreased by 18.14 mg/dl, LDL-cholesterol by10.93 mg/dl and tryglicerides by 49.1 mg/dl at T1. However, it is noteworthy that in spite of the significant LDL-cholesterol reduction – from 105 to 95 mg/dl – many patients did not reach the recommended targets for T2DM (ESC 2021) [32]. Furthermore, non-HDL cholesterol is an established but seldom investigated cardiovascular risk factor [9–12]. Its therapeutic goal is stringent (<  100 mg/dL) in high risk population [6, 7]. In our study, it decreased by 20.75 mg/dl, but at T1 mean level was 125 mg/dL [12]. These findings reinforce the urgent need to intensify lipid-lowering therapy and dietary education, with semaglutide contributing to the achievement of the target.

Important information comes also from the analysis of treatment schemes. The present study substantially involved T2DM patients treated with 1 or 2 OHAs with elevated baseline levels of HbA1c. A small minority of patients were already treated with GLP1-RA and 1 out of 10 patients were treated with insulin before starting semaglutide. This picture underlines the existence of a certain clinical inertia, due, as known, to multifactorial reasons, such as COVID-19 pandemics, long waiting lists, transfer of new patients from other hospitals, low patient adherence, etc.… However, at semaglutide initiation, the proportion of patients treated with insulin and with more than 1 OHA decreased. Even the use of antihypertensive drugs slightly diminished. These data support the most recent evidence on simplification of therapy as a key strategy to overcome clinical inertia [29].

Finally, DTSQ average values documented high levels of satisfaction with treatment (median score was 33 against a maximum score of 36) [15, 16]. Satisfaction with treatment is an important mediator of patient adherence and achievement of targets [38].

Reduced appetite and energy intake, with less preference for energy‐rich foods, were investigated in previous studies and were identified as a possible mechanism to explain the weight loss observed with once-weekly and oral semaglutide [14, 39, 40].

In our setting, we administered a translated and modified version of COEQ adapted for Italian T2DM patients: after 32 weeks of therapy a lower fatty food preference was declared (61%) with a relatively preserved proteic (fish, meat) and carbohydrate rich food (grains, bread, pasta) intake. In another study on 3685 obese subjects, semaglutide induced specific fat mass loss, and energy intake reduction; COEQ questionnaire documented food habits changes with lower craving [18].

In our study, semaglutide significantly improved glucose control and reduced body weight (HbA1c decrease: −1.38%; weight loss: −6.03 kg with 0.5 mg of semaglutide) confirming or even surpassing results of SUSTAIN studies [36, 37, 41]. In addition to drug effect, in our real-world setting, even the attention to the individual dietetic plan, based on Mediterranean Diet model, and dietary/lifestyle education played a role [42]. In the future, it will be interesting to investigate the hepatological impact of this treatment approach in T2DM patients with Nonalcoholic Fatty Liver Disease.

### Strenghts and limitations

The major strength was the inclusion of clinically important but seldom investigated endpoints: non-HDL cholesterol, reduction of antihypertensive drugs prescribed, treatment satisfaction, and eating behaviors. Among limitations, it should be underlined the lack of administration of DTSQ and COEQ at T0 to assess changes over time in the scores and the lack of information about mild hypoglycemia and glycemic variability.

## Conclusion

The study documented benefits of treatment with once-weekly semaglutide on metabolic control and CV risk factors, simplification of therapeutic schemes and high satisfaction with diabetes treatment, and eating behaviors indicative of healthy diet and reduced food intake. GLP1-RAs represent a pivotal drug class that can change favorably the natural history of diabesity.

## Data Availability

The datasets generated during and/or analyzed during the current study are available from the corresponding author on reasonable request.

## References

[1] International Diabetes Federation. IDF Diabetes Atlas. 7th ed. IDF Web site. IDF, 2015. Accessed August 16, 2017

[2] Nauck MA, Keine N, Orscov,  (1993). Normalization of fasting Hyperglycaemia by exogenous glucagon-like peptide 1 in type 2 diabetic patients. Diabetologia.

[3] Raccah D, Chou E, Colagiuri S (2017). A global study of the unmet need for glycemic control and predictor factors among patients with type 2 diabetes mellitus who have achieved optimal fasting plasma glucose control on basal insulin. Diabetes Metab Res Rev.

[4] Flint A, Raben A, Astrup A, Holst JJ (1998). Glucagon-like peptide 1 promotes satiety and suppresses energy intake in humans. J Clin Invest.

[5] Gutzwiller JP, Drewe J, Goke B (1999). Glucagon-like peptide-1 promotes satiety and reduces food intake in patients with diabetes mellitus type 2. Am J Physiol.

[6] Dickson SL, Shirazi RH, Hansson C, Bergquist F, Nissbrandt H, Skibicka KP (2012). The glucagon-like peptide 1 (GLP-1) analogue, exendin-4, decreases the rewarding value of food: a new role for mesolimbic GLP-1 receptors. J Neurosci.

[7] Hjerpsted JB, Flint A (2018). Semaglutide improves postprandial glucose and lipid metabolism, and delays first-hour gastric emptying in subjects with obesity. Diabetes Obes Metab.

[8] Ram N, Ahmed B, Hashmi F, Jabbar A (2014). Importance of measuring Non-HDL cholesterol in type 2 diabetes patients. J Pak Med Assoc.

[9] Eliasson B, Cederholm J, Eeg-Olofsson K, Svensson AM, Zethelius B, Gudbjörnsdottir S, Register ND (2011). Clinical usefulness of different lipid measures for prediction of coronary heart disease in type 2 diabetes: a report from the Swedish national diabetes Register. Diabetes Care.

[10] Brunner FJ, Waldeyer C, Ojeda F (2019). Application of non-HDL cholesterol for population-based cardiovascular risk stratification: results from the Multinational Cardiovascular Risk Consortium. Lancet.

[11] Carr SS, Hooper AJ, Sullivan DR, Burnett JR (2019). Non-HDL-cholesterol and apolipoprotein B compared with LDL-cholesterol in atherosclerotic cardiovascular disease risk assessment. Pathology.

[12] Grundy SM, Cleeman JI, Merz CN (2004). (2016) Implications of recent clinical trials for the National Cholesterol education program adult treatment panel III guidelines. Circulation.

[13] Marso SP, Bain SC, Consoli A (2016). SUSTAIN-6 Investigators. Semaglutide and cardiovascular outcomes in patients with type 2 diabetes. N Engl J Med.

[14] Blundell J, Finlayson G, Axelsen MB (2017). Effects of once-weekly semaglutide on appetite, energy intake, control of eating, food preference and body weight in subjects with obesity. Diabetes Obes Metab.

[15] Bradley C, Bradley C (1994). Diabetes treatment satisfaction questionnaire (DTSQ). Handbook of psychology and diabetes.

[16] Nicolucci A, Giorgino R, Cucinotta D (2004). Validation of the Italian version of the WHO well-being questionnaire (WHO-WBQ) and the WHO-diabetes treatment satisfaction questionnaire (WHO-DTSQ). Diabet Nutr Metab.

[17] Hill AJ, Weaver CF, Blundell JE (1991). Food craving, dietary restraint and mood. Appetite.

[18] Blundell J, Finlayson G, Axelsen M, Flint A, Gibbons C, Kvist T, Hjerpsted JB (2017). Effects of once-weekly semaglutide on appetite, energy intake, control of eating, food preference and body weight in subjects with obesity. Diabetes Obes Metab.

[19] Ahmann A, Chow F, Vivian F, et al (2018) Semaglutide provides superior glycemic control across SUSTAIN 1–5 clinical trials. Int J Nutrol, 11(01):Trab722.

[20] Mann JFE, Hansen T, Idorn T (2020). Effects of once-weekly subcutaneous semaglutide on kidney function and safety in patients with type 2 diabetes: a post-hoc analysis of the SUSTAIN 1–7 randomised controlled trials. Lancet Diabetes Endocrinol.

[21] Brown RE, Bech PG, Aronson R (2020). Semaglutide once weekly in people with type 2 diabetes: real-world analysis of the Canadian LMC diabetes registry (SPARE study). Diabetes Obes Metab.

[22] Jain AB, Kanters S, Khurana R, Kissock J, Severin N, Stafford SG (2021). Effectiveness analysis of switching from liraglutide or dulaglutide to semaglutide in patients with type 2 diabetes mellitus: the retrospective REALISE-DM study. Diabetes Ther.

[23] Lingvay I, Kirk AR, Lophaven S, Wolden ML, Shubrook JH (2021). Outcomes in GLP-1 RA-Experienced patients switching to once-weekly semaglutide in a real-world setting: the retrospective, observational expert study. Diabetes Ther.

[24] Visaria J, Uzoigwe C, Swift C, Dang-Tan T, Paprocki Y, Willey VJ (2021). Real-world effectiveness of once-weekly semaglutide from a US commercially insured and medicare advantage population. Clin Ther.

[25] Rajamand Ekberg N, Bodholdt U, Catarig AM (2021). Real-world use of once-weekly semaglutide in patients with type 2 diabetes: results from the SURE Denmark/Sweden multicentre, prospective, observational study. Prim Care Diabetes.

[26] Hansen KB, Svendstrup M, Lund A, Knop FK, Vilsbøll T, Vestergaard H (2021). Once-weekly subcutaneous semaglutide treatment for persons with type 2 diabetes: real-world data from a diabetes out-patient clinic. Diabet Med.

[27] Di Loreto C, Minarelli V, Nasini G, Norgiolini R, Del Sindaco P (2022). Effectiveness in real-word of once weekly semaglutide in people with type 2 diabetes glucagon-like peptide receptor agonists naïve or switchers from other glucagon-like peptide receptor agonists: results from a retrospective observational Umbria Study. Diabetes Ther.

[28] Zobel EH, von Scholten BJ, Hansen TW (2022). The importance of addressing multiple risk markers in type 2 diabetes: results from the LEADER and SUSTAIN 6 trials. Diabetes Obes Metab.

[29] AMD SID Linea Guida della Società Italiana di Diabetologia (SID) e dell’Associazione dei Medici Diabetologi (AMD). La terapia del diabete mellito di tipo 2. (2021). https://www.siditalia.it/pdf/LG_379_diabete_2_sid_amd.pdf [Italian[. Last access March 3rd, 2022

[30] American Diabetes Association Professional Practice Committee, Draznin B, Aroda VR, et al (2022) 1. Improving care and promoting health in populations: standards of medical care in diabetes-2022. Diabetes Care, 45(Supplement_1):S8-S16 10.2337/dc22-S001 34964872

[31] World Health Organization. Overweight and Obesity. 2018. http://www.who.int/news-room/fact-sheets/detail/obesity-and-overweight. Accessed 10 June 2019

[32] Pintaudi B, Scatena A, Piscitelli G (2021). Clinical profiles and quality of care of subjects with type 2 diabetes according to their cardiovascular risk: an observational, retrospective study. Cardiovasc Diabetol.

[33] Nicolucci A, Rossi MC (2008). Incretin-based therapies: a new potential treatment approach to overcome clinical inertia in type 2 diabetes. Acta Biomed.

[34] Iqbal J, Wu HX, Hu N (2022). Effect of glucagon-like peptide-1 receptor agonists on body weight in adults with obesity without diabetes mellitus-a systematic review and meta-analysis of randomized control trials. Obes Rev.

[35] Harder H, Nielsen L, Tu DT, Astrup A (2004). The effect of liraglutide, a long-acting glucagon-like peptide 1 derivative, on glycemic control, body composition, and 24-h energy expenditure in patients with type 2 diabetes. Diabet Care.

[36] Pratley RE, Aroda VR, Lingvay I (2018). Semaglutide versus dulaglutide once weekly in patients with type 2 diabetes (SUSTAIN 7): a randomised, open-label, phase 3b trial. Lancet Diabet Endocrinol.

[37] Capehorn MS, Catarig AM, Furberg JK (2020). Efficacy and safety of once-weekly semaglutide 1.0mg vs once-daily liraglutide 1.2mg as add-on to 1–3 oral antidiabetic drugs in subjects with type 2 diabetes (SUSTAIN 10). Diabet Metab.

[38] Khunti K, Millar-Jones D (2017). Clinical inertia to insulin initiation and intensification in the UK: a focused literature review. Prim Care Diabet.

[39] Davies M, Speight J (2012). Patient-reported outcomes in trials of incretin-based therapies in patients with type 2 diabetes mellitus. Diabetes Obes Metab.

[40] Gibbons C, Blundell J, Tetens Hoff S, Dahl K, Bauer R, Baekdal T (2021). Effects of oral semaglutide on energy intake, food preference, appetite, control of eating and body weight in subjects with type 2 diabetes. Diabetes Obes Metab.

[41] Ahmann AJ, Capehorn M, Charpentier G (2018). Efficacy and safety of once-weekly semaglutide versus exenatide ER in subjects with type 2 diabetes (SUSTAIN 3): a 56-week, open-label, randomized clinical trial. Diabet Care.

[42] Evert AB, Boucher JL, Cypress M (2014). Nutrition therapy recommendations for the management of adults with diabetes. Diabet Care.

